# A supervised scheme for aspect extraction in sentiment analysis using the hybrid feature set of word dependency relations and lemmas

**DOI:** 10.7717/peerj-cs.347

**Published:** 2021-02-05

**Authors:** Bhavana R. Bhamare, Jeyanthi Prabhu

**Affiliations:** 1 Department of Computer Science and Engineering, Sathyabama Institute of Science and Technology, Chennai, Tamilnadu, India; 2 Department of Information Technology, Sathyabama Institute of Science and Technology, Chennai, Tamilnadu, India

**Keywords:** Feature extraction, Aspect based sentiment analysis, Machine learning, Natural language processing, Support vector machine

## Abstract

Due to the massive progression of the Web, people post their reviews for any product, movies and places they visit on social media. The reviews available on social media are helpful to customers as well as the product owners to evaluate their products based on different reviews. Analyzing structured data is easy as compared to unstructured data. The reviews are available in an unstructured format. Aspect-Based Sentiment Analysis mines the aspects of a product from the reviews and further determines sentiment for each aspect. In this work, two methods for aspect extraction are proposed. The datasets used for this work are SemEval restaurant review dataset, Yelp and Kaggle datasets. In the first method a multivariate filter-based approach for feature selection is proposed. This method support to select significant features and reduces redundancy among selected features. It shows improvement in *F*1-score compared to a method that uses only relevant features selected using Term Frequency weight. In another method, selective dependency relations are used to extract features. This is done using Stanford NLP parser. The results gained using features extracted by selective dependency rules are better as compared to features extracted by using all dependency rules. In the hybrid approach, both lemma features and selective dependency relation based features are extracted. Using the hybrid feature set, 94.78% accuracy and 85.24% *F*1-score is achieved in the aspect category prediction task.

## Introduction

Quick improvements in e-commerce websites lead customers to purchase and analyze products online. Also, it allows end-users to express their views/opinions related to an item and services by means of reviews. These opinions are useful for other users to decide about the purchase of a product. These are also helpful to manufacturers to enhance the quality of their items and services and they may know what exactly customers want. In any case, it is hard for an individual to analyze a large number of reviews and rate them according to various aspects of the product. Hence, it is required to analyze all users’ views and classify them with respect to different aspects.

Sentiment analysis (SA) has a significant role in analyzing and summarizing all the opinions. SA is the analysis of the reviews given by the people about any product on various e-commerce websites or social media, etc. SA can be done at different levels of granularity (Hu & Liu, 2004), they are aspect, sentence and document level. Aspect level SA recognizes the polarity of each individual aspect of a product. It includes tasks like aspect term extraction and opinion target extraction etc.

In sentence level SA, polarity is predicted for the complete sentence. It deals with the recognition of statements as objective or subjective, while in document level SA the polarity is predicted for the complete review or document. It extracts opinion bearing words and detects its polarity. The work in this paper focuses on ABSA.

Aspect is nothing but the component or attribute of the product. In other words, ABSA is a SA method that finds the aspects/attributes of a product and afterward designates an estimation level (positive, negative or neutral) to each attribute. The large distinction between SA and ABSA is that the former just distinguish the feeling of full text, while the latter breaks down every content to recognize different aspects and decide the relating sentiment for each one of them. Aspects can be implicit or explicit based on the presence of aspect terms. Statements with implicit aspects do not contain direct aspect terms. Instead, we need to recognize it from the words or expressions expressed in the user reviews. The following two sentences are reviews about mobile phones. For a mobile phone, aspects can be a battery, camera, audio, memory, processing speed, etc. In sentence 1, the aspect term is a camera and the sentiment specifying word is “good” that is, sentiment is positive. In this sentence, the camera word is not used directly instead it is signified by the phrase “picture quality”. So the aspects may be specified explicitly or implicitly in the sentence. The implicit aspect in the second sentence is a battery which is represented by the word “charging”.

Sentence 1: The picture quality of this mobile is good.
Sentence 2: It does not need charging for a long time.

This section focuses on exhaustive literature review which covers various aspects of contemporary area of research. The survey focuses on ABSA applications, aspect extraction and selection techniques suggested in earlier works, knowledge-based approaches to consider semantic features, topic-based methods for selecting features that are independent of the subject matter and deep CNN approach. ABSA analysis has a number of applications such as movie rating prediction, restaurant recommendation, financial news analysis (Ahmad, Cheng & Almas, 2007), political predictions, etc. Benkhelifa, Bouhyaoui & Laallam (2019) proposed a system that will rank numerous cooking recipes which helps to choose the finest by using reviews of users. This system also makes use of metadata of YouTube videos and improves the performance. It has performed three tasks subjectivity detection, aspect extraction, and sentiment classification. For both subjectivity detection and sentiment classification, features are selected using TF-IDF feature weighting method and for classification NB and SVM classifiers are used. In both, the SVM algorithm outperforms the NB classifier. The aspect extraction is done with a rule-based approach using Stanford dependency parser. Akhtar et al. (2017) proposed a recommender system that endorses the best hotel for the user. In this approach, first, the topic modeling technique is used to recognize concealed data and aspects. Further, the sentiment analysis is used on classified sentences and finally, summarization is done. For topic modeling, the MALLET tool is used and sentiment analysis is done using the SentiWordNet corpus. As reviews are summarized based on aspect category, it gives a better understanding of the reviews. Afzaal, Usman & Fong (2019) proposed a framework for mobile tourism app using ABSA. With POS tagger, the nouns and noun phrases are extracted seeing them as candidate features to decide aspect category. The co-referential aspects are clustered by means of WordNet and aspects with occurrence count more than 10 are selected that assisted to extract explicit aspects. A decision tree approach is used to pull out implicit aspects. Sentiment analysis is done using five different classifiers, amongst all NBM classifier shown good results with an accuracy of 88.08% on the restaurant review dataset. Vanaja & Belwal (2018) and Anand & Naorem (2016) analyzed Amazon customer surveys and Amazon movie reviews, respectively. Vanaja & Belwal (2018) distinguished among Naïve Bayes and SVM algorithm on sentiment analysis task. In it, features are extracted by applying part of speech tagging and selecting nouns, verbs, adverbs, adjectives, and pronouns from each review. Frequent features are selected using the Apriori algorithm. These features are pruned by removing stop words and then the classifiers are applied to determine the class labels as positive, negative or neutral. The NB classifier outperformed the SVM with accuracy 90.423%. Anand & Naorem (2016) performed ABSA in two stages: to detect aspect and determine sentiment for the aspect. The Stanford dependency parser is applied using some handcrafted rules to extract aspect-sentiment pairs. The polarity of extracted sentiment words is determined using the SentiWordNet corpus. To detect the aspect some aspect clue words are used. These clue words are chosen by three approaches: manual labeling, clustering, and review guided clustering. El Hannach & Benkhalifa (2018) proposed crime identification using implicit aspects and it is done on the twitter dataset. This research is carried out in three main stages: implicit aspect sentence detection, implicit aspect term extraction and implicit aspect identification (IAI). The features used for this work are adjectives and verbs along with its WordNet synonyms selected using Term Frequency-Inverse Class Frequency (TF-ICF). The classifiers used for IAI are MNB, SVM and RF. This work has shown that the usage of TF-ICF achieves better compared to TF-IDF.

ABSA can be done using different approaches. Many approaches focus on feature extraction and selection process. Features can be selected using strategies like occurrence frequency, syntax-based rules, semantics or the hybrid approach. Significant features are more supportive to predict the aspect category and sentiment class. A comparative study of numerous existing language rule-based aspect extraction methods is quantified by Ruskanda, Widyantoro & Purwarianti (2018). Observations demonstrate that the accuracy of a language rule-based aspect extraction technique is broadly resolved by the completeness and accuracy of rules compared to other technologies. Poria et al. (2014) proposed a dependency rule-based methodology to resolve the issue of aspect extraction from reviews of products. In this research, authors used Stanford parser to get the dependency tree and further some dependency rules are applied to mine aspects from a sentence. This work indicates significant improvement as it works on the extraction of relevant features. Rana & Cheah (2017a), Rana & Cheah (2017b) and Rana & Cheah (2019) worked on ruled based methods for extracting product features. Rana & Cheah (2017a) presented a two-fold rule-based approach to extract aspects. In this, first, fold extract aspects related to domain-independent opinions and second fold extract aspects related to domain-dependent opinions. The author also applied frequency and similarity measure to enhance the accuracy of the aspect extraction of an established system. Rana & Cheah (2017b) proposed a two-level aspect pruning approach that can reduce inappropriate aspects. The author used the sequential pattern-based model to extract noun phrases that represent aspects. Aspect elimination is done by estimating the frequency of each word and picking the most frequent aspects. Further, the semantic similarity measure is used to select non-frequent features. Asghar et al. (2019) proposed a framework performing aspect extraction, sentiment classification, and summary generation. In this work, heuristic rules are used for aspect-sentiment pair extraction. The aspect terms are grouped based on their co-reference PMI value and assigned one aspect category. The sentiment classification is done using the SentiWordNet lexicon. In this work, a summary is generated as a list of positive and negative aspects individually with their sentiment scores. Shafie et al. (2018) presented research that uses numerous types of dependency relation for extracting candidate aspects from user review. Firmanto & Sarno (2019) proposed an aspect-based sentiment analysis method utilizing grammatical rules, word similarity and SentiCircle. The proposed method starts with the extraction of candidate aspects using grammatical rules. Authors used word embedding and word similarity in preprocessing steps for aspect categorization. For keyword extraction, TF-ICF is used and in the end, SentiCircle is utilized to find sentiment polarity. Agarwal et al. (2015) presented a concept based approach using dependency rules. In this research, the authors used some dependency rules for feature extraction. The extracted features are enriched by adding some commonsense knowledge extracted from ConceptNet. These features are pruned by means of the mRMR feature reduction approach and sentiment classes are predicted using an SVM classifier. Asghar et al. (2017) used a rule-based classification system to enhance the sentiment classification of reviews in online communities. The essential purpose of this work is to improve the overall performance of sentiment analysis by considering additional features like modifiers, emoticons, domain-related phrases and negations. Kang & Zhou (2017) proposed RubE—unsupervised rule-based techniques to extract subjective and objective features from online consumer reviews. In this work, objective features are collected by integrating part-whole relations and patterns that are specific to reviews. Subjective features collected by applying double propagation with indirect dependency and comparative construction.

In ABSA, after feature extraction, feature pruning is necessary to avoid the risk of overfitting and improve accuracy. Reduced features require less time for training. The statistical weight of features can be used to reduce the feature set. So selecting or proposing a feature selection strategy is an open research issue. Manek et al. (2017) presented a Gini Index based feature selection technique with SVM classifier that classifies sentiment of movie reviews. This method verified that the Gini Index improved classification accuracy compared to other feature weighting techniques. Liu et al. (2018) proposed a Weighted Gini Index (WGI) feature selection method for imbalanced data. Various algorithms namely Chi-square, F-statistic and Gini index feature selection are compared with proposed system. According to their work F-statistic provides the best performance in the minority class. If the numbers of selected features are more, WGI feature selection lead to better performance. Uysal (2016) proposed an enhanced global feature selection system which enhances the classification performance. It offers an improved approach to filter-based feature selection. The Improved Global Feature Selection Scheme (IGFSS) is the combination of the global feature selection technique and the local feature selection method. The performance of classification is significantly improved with the IGFSS approach.

Many researchers merged commonsense knowledge, the semantics of features with ontology to improve the accuracy of aspect extraction and sentiment classification. Schouten, Frasincar & de Jong (2017) and Schouten & Frasincar (2018) proposed ontology-enhanced aspect-based sentiment analysis. Schouten, Frasincar & de Jong (2017) concentrated on a knowledge-driven solution with the aim to complement standard machine learning (ML) method. The writers encoded the domain knowledge into a knowledge repository/ontology. In this research, both aspect detection and aspect sentiment analysis is done. It shows that the embedding of commonsense knowledge with ontology-based features improve the performance of classification. For both tasks the libsvm classifier is used. Schouten & Frasincar (2018) prepared ontology with three classes like SentimentMention, AspectMention, and Sentiment Value. The ontology is generated using an onto-clean methodology. If the ontology does not predict any class, then the class prediction is done using the Bag-of-Words backup model. This research signifies that encoding domain knowledge in ontology advances the result of sentiment classification. De Kok et al. (2018a) and De Kok et al. (2018b) proposed an ontology centered approach for review level aspect-based sentiment analysis. In this work, they mainly focus on ontology-enhanced methods that complement a standard ML algorithm. For these two different algorithms are used, a review-based and a sentence aggregation algorithm. Their work contains feature generators and feature adaptors. Several feature generators are independent of the ontology which are aspect, sentence count, lemma and several are dependent on an ontology which are ontology concepts, sentiment count. Also, they used several feature adaptors which are ontology concept score, negation handling, synonyms, weight, word window, etc. Ma, Peng & Cambria (2018) improved the LSTM network with a hierarchical attention mechanism. The main influence of this work is the integration of commonsense knowledge of sentiment related concepts into an attentive LSMT. This research accomplished the two tasks that is, aspect extraction and sentiment classification.

Zeng et al. (2019) used a convolutional neural network (CNN) with linguistic resources and gating mechanism for aspect-based sentiment analysis (ABSA). Al-Smadi et al. (2019) suggested a supervised learning method for aspect extraction and classification of sentiments. The classifiers are trained on lexical, morphological, syntactic and semantic features. Baas et al. (2019) introduced a new approach by using SVM with six different pattern classes. This includes lexical, syntactic, semantic, hybrid, and surface feelings. The lexico-semantic patterns were used in the customer reviews to identify aspect-based feeling (ABS). Synset bigram, negator POS bigram, and POS bigram are used to enhance the extraction of aspects based on feelings.

Latent Dirichlet Allocation topic model is applied by Amplayo, Lee & Song (2018). This work is an extension of the Aspect and Sentiment Unification Model (ASUM). It considers seller sentiments for aspect extraction and sentiment classification. Seller-aided Aspect-based Sentiment Model (SA-ASM) and Seller-aided Product based Sentiment Model (SA-PBM) are proposed. SA-ASM provides improved results for sentiment classification and SA-PBM for aspect extraction. Al Moubayed, McGough & Hasan (2020) proposed a deep learning approach for topic modelling. Topic modelling is used in this method for extraction of features and it eliminates the need for subject-specific lexicon. Firstly, the dataset is categorized into two categories like positive and negative. Then topic modelling is used to extract features from each class of training dataset that are further given as input to train the stacked denoising autoencoders (SDAs). The overall reconstruction error from each SDA is used by a linear classifier to predict the class.

Xu et al. (2019) suggested a new implicit aspect recognition method that relies on non-negative matrix factorization (NMF). This method clusters aspects of a product by merging co-occurrence data with intra-relations of aspects and sentiments. Kim (2018) proposed an advanced semi-supervised system for reducing dimensionality. Weighting and extraction of features are performed in a semi-supervised manner. Kumar, Pannu & Malhi (2020) introduced the aspect based SA using semantic features and deep networks. Domain-specific ontology is generated after preprocessing of review sentences to obtain semantic features. The Word2vec is generated by applying unsupervised neural network to the processed corpus. This vector is used for training CNN model. This method has produced significant results. The CNN model is optimized using particle swarm optimization.

The subtasks in ABSA are aspect term extraction, aspect category detection, opinion term extraction, and sentiment analysis. The proposed system is focusing only on aspect category detection. In aspect category detection task aspect terms are important to detect category. If the aspect terms are not specified explicitly then it can be predicted from the opinion words also. Therefore, the proposed system works on lemma features as well as dependency rule-based features. Dependency rule-based features are meaningfully related words in a sentence which support to predict aspect category. The main objectives of this research work are, (i) to examine the effect of feature selection strategy on classification performance, (ii) to examine the effect of dependency rule-based features on the classification performance. The datasets used in this system are SemEval 2014 restaurant review dataset (Pontiki et al., 2016), Yelp and Kaggle datasets. The article is structured as; “Introduction” highlights recent developments in the field of research, proposed method is presented in “Proposed Method”, “Results and Discussion” focuses on results and discussion and concluding remarks is presented in “Conclusions”.

## Proposed Method

**Figure 1:**
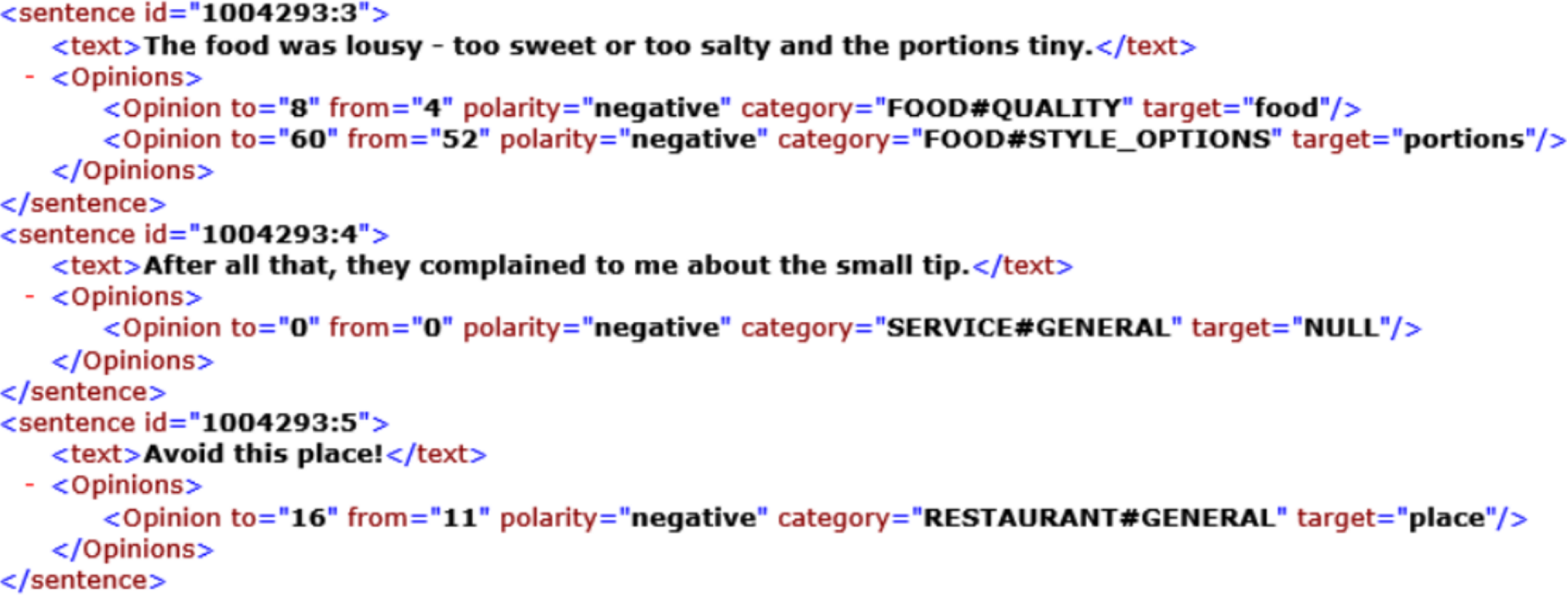
Example snippet from restaurant review dataset.

**Figure 2:**
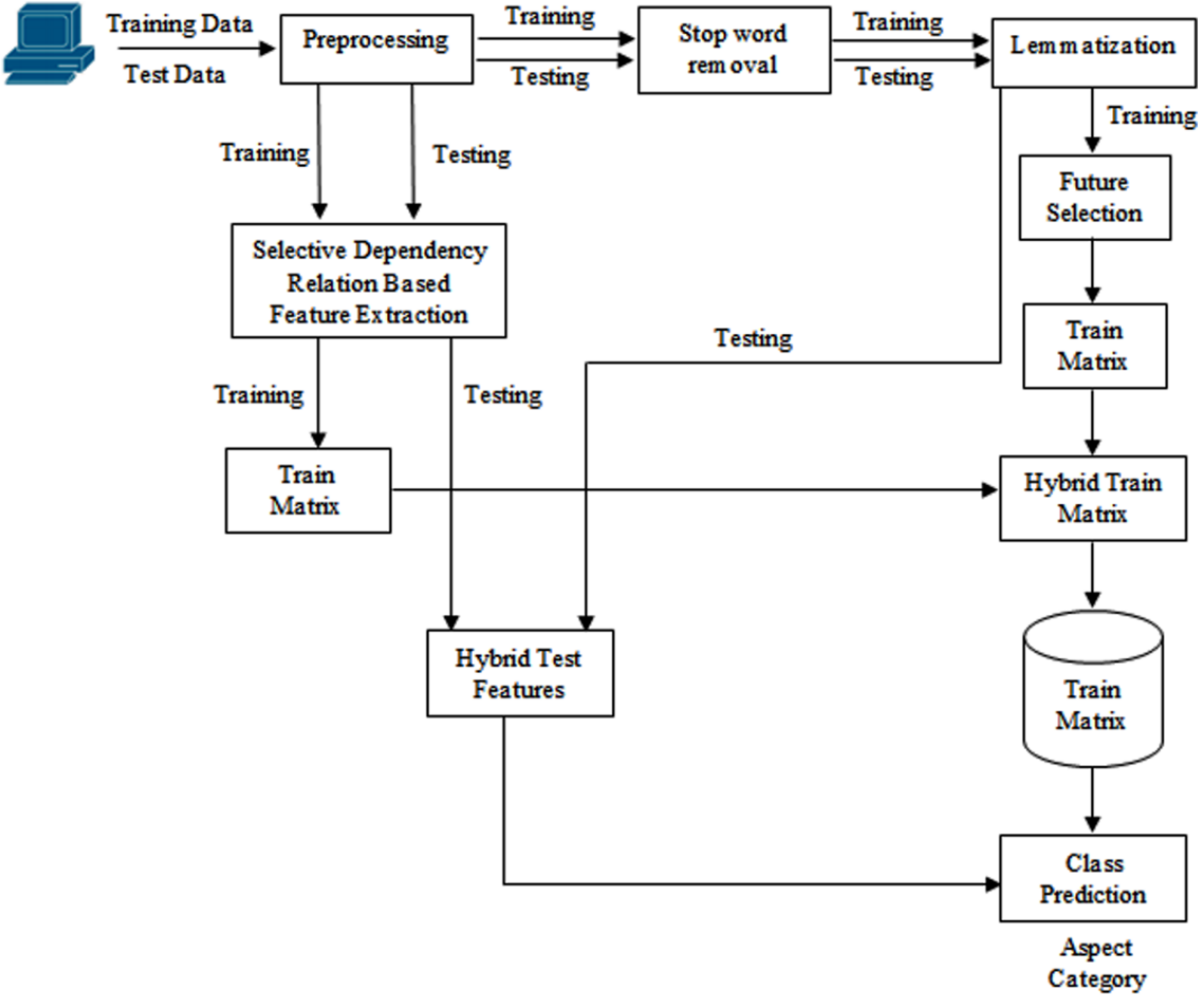
Proposed system architecture.

The technique presented in this article uses a supervised methodology for aspect extraction from review sentences. The datasets used for this research are SemEval 2014 restaurant review dataset, Yelp and Kaggle datasets. SemEval restaurant review dataset contains 3,044 training sentences and 800 test sentences. The restaurant reviews are specified in sentence format. The snippet of the dataset is shown in Fig. 1. This dataset has sentences with the aspect categories like food, ambiance, price, service and miscellaneous/anecdotes. These categories of aspects can be specified explicitly or implicitly. Explicit aspect categories are specified directly in the sentences. For example, in the above snippet, the first sentence is about the food aspect and the aspect is explicitly specified in it. The aspect of the second review sentence is service, but it is not specified explicitly. The word “small tip” specifies it. In reviews, a single sentence may have one or more aspect categories. The aim of this study is to extract aspects from the review sentences and not to decide sentiments. The detailed architecture is demonstrated in Fig. 2. The process of aspect category extraction is elaborated as below.

### Preprocessing

First, the stop words are removed from training and test sentences and then the lemmatization is done. From both datasets, punctuation marks are removed and contractions like can’t, isn’t, etc. are replaced with cannot and is not.

### Feature extraction

In feature extraction, two types of features are extracted from the training dataset and a hybrid feature set is created. The extracted features include:

- Lemma features
- Rule based features
- Hybrid features

A) *Lemma features:* The process of lemma feature extraction and selection includes the following steps:

1. After preprocessing, the lemmas are extracted from each sentence. A matrix is generated from these lemma features that contain review id, lemma feature, term frequency of each feature and aspect category label. Here, in the corresponding aspect group, term frequency is the number of times the term is present in that aspect category. From this matrix, distinct lemmas with term frequency more than threshold *thf* in corresponding aspect category are selected. Here, threshold *thf* is 3. This value is selected based on intuition.
2. Further, a matrix is generated which contains review id, lemma features, its term frequency in each aspect categories and the actual aspect category label.
3. This matrix is considered as a training matrix.

During testing, the following process is followed.

1. From each test sentence, lemmas are extracted.
2. For each lemma in the test sentence, a vector from a training matrix for the matching lemma is copied to the test matrix.
3. The above process is done for all lemma features in a test sentence. Then probability is calculated for each aspect category. The aspect category having the highest probability is given as a category label for the test sentence.

In line with the first objective, the above is the base system where features are selected using term frequency only. The same experimentation is carried out using a multivariate filter method of feature selection.

In classification problems, an excessive number of features not only increase the computational time but also degrade the accuracy of classification (Abualigah et al., 2017). So it is necessary to extract significant features. These selective features train the machine learning algorithm faster, increases accuracy and avoids overfitting. Methods of feature selection can be classified into categories: filter, wrapper, and hybrid approaches (Labani et al., 2018). In the wrapper method of feature selection, a subset of features is selected to train the machine learning algorithm. Based on the results, the features are added or removed from the set. In the filter approach, feature selection is independent of any machine learning algorithm. Here, features are selected based on statistical tests like Linear discriminant analysis, Analysis of variance, Chi-square etc. Filter methods are classified as a univariate and multivariate feature selection methods. In the univariate method, individual features are assigned a statistical score and top-ranked features are selected. The disadvantage of this approach is that it selects relevant features and ignores redundancy among them as it ignores the relationship between features. In the multivariate method, the relationship between individual features is considered. The combination of filter and wrapper methods is the hybrid method.

The proposed system uses a multivariate filter approach. In it, relevant features are selected by means of weighted term frequency score and redundancy is avoided by calculating the Pearson correlation coefficient between features. In the proposed system, similar to the base system, features having term frequency greater or equal to three is selected. Weight is calculated for the selected terms as per Eq. (1). Terms with a weight greater than threshold *th*_wk_ are selected. Here *th*_wk_ is the threshold on weight in aspect category *k*. From these features, a matrix is generated which contains review id, feature, term frequency in all aspect categories and the actual aspect category label. For each feature in an aspect category, the correlation is determined with all other terms in the related category. Features with correlation value that exceeds the threshold *thc* are not considered because these are highly correlated features that may increase redundancy. The value of threshold *thc* for this experimentation is 0.85 which is selected through repetitive testing. Similar to the base system, a training matrix is generated using selected features and testing is performed.

(1) $${\rm weight}\;\left( f \right) = \displaystyle{{{\rm frequency}\;\left( f \right)k} \over {{\rm total\;frequency}\;\left( f \right)}}$$

The above-stated feature selection strategy enhanced the result of the aspect extraction task compared to the method which uses term frequency-based features only. This feature selection approach is an extension of the work described in Bhamare, Jeyanthi & Subhashini (2019) where a correlation was calculated using the weight of terms. Here it is calculated using the term frequency of features.

B) *Rule based features:*

In this approach, features are selected based on grammatical relationships between words. Stanford NLP parser is used to extract grammatical relationships between words. Sometimes the phrases or related words in a sentence give more information about the aspect compared to the single lemma features.

**Figure 3:**
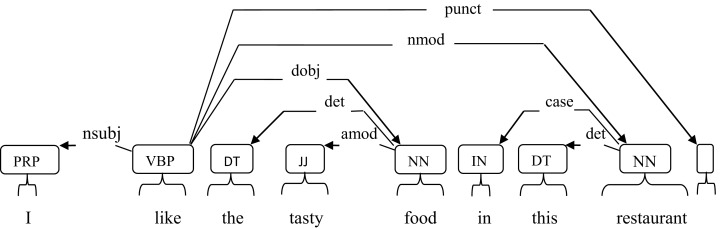
Dependency relations in a sentence.

As in Fig. 3, the arrows indicate the relationship between words in a sentence. The parser extracts the features: like restaurant, like food, like I, restaurant this, food tasty, etc. In the first testing, all the dependency relationships except determinant (det) relation are used to extract rule-based features. For each pair of features in an aspect category, its term frequency is calculated and distinct features are selected. Here, term frequency is not applied for feature selection as many dependency relations will not occur regularly. From these selected features, a matrix is generated which contains review id, feature, its term frequency in each aspect category and the actual aspect category label. This matrix is the training matrix. Testing is performed similar to lemma based approach but the difference is that from each test sentence instead of lemma features rule-based features are extracted.

In the second experimentation, features are not selected by considering all grammatical rules. Here, the dependency relations which contain any of the nouns, adjectives, adverbs are used to extract features. The rules used are mentioned below (De Marneffe & Manning, 2010):

**acomp:** adjectival complement

In a sentence, when an adjectival phrase acts as a complement to the main verb in the sentences it is defined as an adjectival complement.

**advcl:** adverbial clause modifier

In a complex sentence, the clause modifying the relationship of the verbal phrases (viz. temporal, conditional, comparative, purpose clause, concessional, manner and result) is identified as an adverbial clause modifier.

**advmod:** adverb modifier

In a sentence or phrase an adverb modifier can be defined as a word that modifies a noun, an adjective or a verb; especially in a non-clausal sentence.

**agent:** agent

An agent is a word that complements the passive verb in a sentence and is explained further by the use of the preposition “by”. Unlike basic dependencies output, this association is present only in sentences where collapsed dependencies appear.

**amod:** adjectival modifier

In a sentence or a phrase, when an adjectival phrase alters the meaning of the noun phrase, it is called as an adjectival modifier.

**conj:** conjunct

A conjunct explains the association between two words that are related and connected by using the co-ordinating conjunctions like “and”, “or”, etc. The conjuncts are treated unevenly; wherein the first conjunct leads and the other conjuncts are dependent on the first by way of relation.

**cop:** copula

A copula is a connecting word, particularly a form of a verb that connects the subject and the complement. Copula is often considered to be dependent on its complement.

**dobj:** direct object

A direct object is a noun, noun phrase, or pronoun that signifies what or who receives the action of a transitive verb in a clause or sentence.

**neg:** negation modifier

A negation modifier identifies the connection between a negation word or phrase and the NP or VP it modifies.

**nn:** noun compound modifier

A noun compound modifier refers to a noun in the sentence that provides a better understanding of the head noun and tends to modify it.

**nsubj:** nominal subject

A noun phrase is a pro-agent of a clause in a sentence which is also known as a syntactic subject is called a nominal subject. The association in the sentence; unlike standard grammar rules, isn’t controlled by the verb. When there is a copular verb, the root of the clause is the complement of the copular verb which could either be an adjective or a noun.

**nsubjpass:** passive nominal subject

When a nominal subject; which is the syntactic subject, is used in a passive voice in a sentence, it is defined as a passive nominal subject.

**rcmod:** relative clause modifier

A relative clause that modifies the noun phrase is known as a relative clause modifier. It is placed next to the noun it modified but can also be separated wherein an essential modifier is used. The association moves from the noun phrase to the relative clause; usually by use of a verb.

**xcomp:** open clausal complement

A predicative or clausal complement (without its own subject) of a verb or an adjective is known as an open clausal complement. The subject in this case is indicated by phrase / words external to the xcomp. The clausal complements are not adjuncts or modifiers and are always non-finite.

**nmod:** Nominal modifier

The relationship between the noun / predicate modified by the prepositional supplement and the noun introduced by the preposition is the nmod relation.

The testing proves that the system which uses features extracted using selective dependency relations increases the performance compared to the system that uses all dependency relations.

C) *Hybrid features:*

In this method, both lemma features and rule-based features are used to obtain a training matrix. The rule-based features are extracted using dependency relations mentioned in section B and Lemma features are selected using the multivariate filter method. The training matrices of both types of features are concatenated to obtain a single training matrix. At the time of testing, from each sentence, both lemma features and rule-based features are extracted and a similar testing process as mentioned for the base system is followed to determine the aspect category label. This experimentation shows that the performance of aspect category extraction is improved if rule-based features are combined with lemma features. Algorithms 1–3 explain the detail process of the proposed hybrid method.

**Algorithm 1:** Feature selection using multivariate filter method.

Preprocessing: stop word removal and stemming is done for all training samples.
**Input: **Frequency[*i*][*j*]_*k*_ is the matrix of features in aspect category *k* containing the occurrence count (TF) *j* of feature *i*. Threshold for TF is *th*_*f*._ Threshold for correlation *th*_*c*._
**Output:** A Training matrix.
**Step 1:** for every feature *f* in Frequency[ ][ ] if (Frequency[*f*]$\ge t{h_f}$ RFrequency.add(*f*) end for All the selected distinct/unique features are then added to UFrequency[ ][ ] **Step 2:** for every feature *f* in UFrequency weight(*f*)= UFrequency[*f*][*j*]/total[*f*] where total[*f*] is TF value of that feature in all aspect categories and UFrequency[*f*][*j*] is the TF value in that aspect category. if weight(*f*) >*th*_*wk*_ where *th*_*wk*_ is a threshold on weight in aspect category *k*. add *f* in weighted[*f*][*j*] where weighted[*f*][*j*] represents the TF of feature *f* in aspect category *j. j* represents aspect categories 1..k. end if end for The weight threshold is different for each aspect category. **Step 3:** A matrix weighted[*i*][*j*] is reorganized with *i* = {*t1, . . ,tn*} and *j*={*ak1, . . ,ak5*} representing 5 aspect categories. Individual row in weighted[*i*][*j*] is TF of feature *i* in *ak1 . . ak5* aspect categories. **Step 4:** for every *ti* in weighted, compute correlation with other features in aspect category *ak* $cor\left[ t \right] = \displaystyle{{n\left( {\sum {X\left[ {\;} \right]} Y\left[ {\;} \right]} \right) - \left( {\sum {X\left[ {\;} \right]} } \right) \times \left( {\sum {Y\left[ {\;} \right]} } \right)} \over {\sqrt {\left[ {n\sum {{X^2}} - {{\left( {\sum X } \right)}^2}} \right]\left[ {n\sum {{Y^2}} - {{\left( {\sum Y } \right)}^2}} \right]} }}$ end for Compute average correlation for each term and update it in weighted[*i*][*j+1*]. **Step 5:** To avoid redundancy, features are selected based on correlation. for every *t* in weighted [*i*][*j+1*] cor[*t*]=weighted[*i*][*j+1*] if (${\rm cor}\left[ t \right] \le {\rm t}{{\rm h}_{\rm c}}$) then Copy row weighted[*t*][*j*] to trainmatrixL[*t*][*l*] end if end for trainmatrixL[][] is the training matrix.

**Algorithm 2:** Feature extraction based on dependency rules.

**Input:** Training dataset **Output:** A training matrix
**Step 1:** For every sentence S in training dataset D Extract grammatical rule based features using Stanford NLP parser **Step 2:** For every rule based feature, find its occurrence count in corresponding aspect category. **Step 3:** For each aspect category, prepare matrix containing distinct rule based features with its occurrence count in that aspect category **Step 4:** Prepare matrix dptrainmatrixL[*i*][*j*] of all rule based features, where *i* represents the feature/term and *j* represents its occurrence count in aspect categories {*ak1,..,ak5*} and the actual category label.

**Algorithm 3:** Algorithm for aspect category extraction using hybrid features.

Preprocessing: stop word removal, stemming applied for all test samples. From test samples, punctuation marks are removed and contractions are replaced with words.
**Input:** trainmatrixL[i][j], dptrainmatrixL[m][n], test dataset **Output:** Aspect label to test sentences.
**Step 1:** Generate hybrid training matrix hybridmatrix[*i*][*j*] ← concat(trainmatrixL, dptrainmatrixL)
**Step 2:** Extract lemma and rule based features from individual test sentence **Step 3:** for every sentence *k* from test dataset for every feature *f* of sentence *k* for every term *t* in hybridmatrix[*t*][*j*] if test feature *f* = = term in hybridmatrix test[*f*][*1..5*]=hybridmatrix[*t*][*1..5*] end if end for end for end for **Step 4:** Calculate the probability of an individual aspect group. **Step 5:** Aspect group with the maximum probability value is returned as a test sentence label.

## Results and Discussion

**Figure 4:**
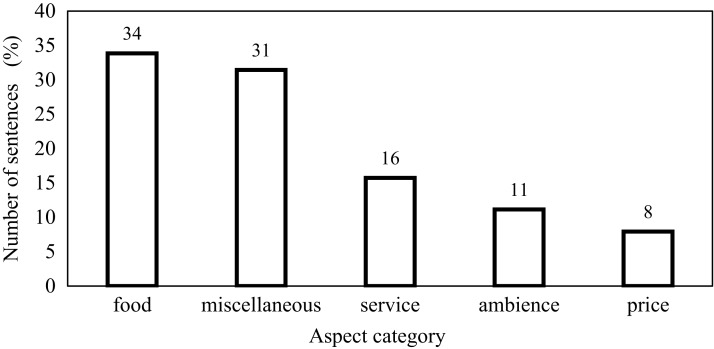
Relative percentage of number of sentences in individual category.

**Figure 5:**
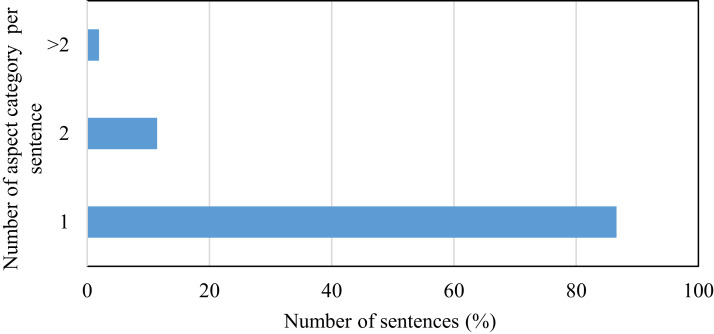
Representation of number of aspect categories per sentence.

This research is carried out on datasets such as the restaurant review dataset SemEval, Yelp and Kaggle datasets. For SemEval dataset, Fig. 4 shows the relative percentage of the total number of sentences in the individual aspect category. The distribution of data between the aspect categories in the dataset is not balanced. This affects the performance of the classifier. In this dataset, 34% of sentences are of the food aspect category and that of price category is only 8%. To handle this problem, features are not extracted by considering the dataset as a whole because it will cause selecting features from major categories only. In the proposed work, review sentences are grouped according to the aspect category and then applying the proposed method, features are extracted from each aspect category. This causes to select relevant features from each aspect category. Some of the sentences in the SemEval dataset have more than one aspect category. Fig. 5 represents this distribution. 87% of sentences have unique aspect categories, 11% have two aspect categories in a sentence and 2% have more than two aspect categories in a sentence. The proposed system extracts only one aspect category from each sentence. In the training and testing dataset, sentences having multiple aspect categories are duplicated and assigned unique categories.

The evaluation measure used in this experimentation is *F*1-score to compare results of different tests. The *F*1 score (Eq. 2) is calculated from precision and recall. In the Eq. (3) and (4) TP is true positive, FP is false positive, and FN is false negative.

(2) $$F1 = 2 \times \displaystyle{{{\rm precision} \times {\rm recall}} \over {\rm {precision + recall}}}$$

(3) $${\rm precision = \displaystyle{{TP} \over {TP + FP}}}$$

(4) $${\rm recall = \displaystyle{{TP} \over {TP + FN}}}$$

Table 1 shows the *F*1 score obtained when only lemma features are used. As for lemma features, two feature selection strategies are used: term frequency-based feature selection approach which is a base system and multivariate filter-based feature selection approach. The results demonstrated the first objective that the feature selection approach improves classification performance. Table 1 shows that the multivariate filter method of feature selection has gained more *F*1 score in all aspect categories compared to the base system. This strategy of feature selection selected relevant features and avoided redundant features whereas the base system focuses on only relevant features and ignores redundancy among features.

**Table 1:** *F*1 score (%) obtained using term frequency based feature selection approach and multivariate filter feature selection approach (SemEval Dataset).

Approach	Aspects	Precision (%)	Recall (%)	*F*1-score (%)
Term frequency approach of feature selection	Ambience	75.51	67.89	71.50
	Miscellaneous	66.78	81.12	73.26
	Food	85.09	80.54	82.75
	Price	81.36	67.61	73.85
	Service	77.12	74.68	75.88
Multivariate filter approach of feature selection	Ambience	81.31	79.82	80.56
	Miscellaneous	81.58	79.83	80.69
	Food	88.30	93.67	90.91
	Price	87.10	76.06	81.20
	Service	87.25	82.28	84.69

Table 2 shows *F*1-score obtained using approach B. In approach B features are selected using dependency relations. In the first experimentation of approach B, features are extracted by considering all dependency relations. In second, features are extracted by applying selective dependency relations. If all dependency relations are considered to extract features, then it increases redundant features. Also, some irrelevant features get extracted which depreciates the performance of classification. The dependency relations considered here are selective which helps to choose relevant features. Another objective of this research is to analyze the effect of dependency rule-based features on the classification performance. This testing proves that features extracted using selective dependency relations improve the performance of classification.

**Table 2:** *F*1- score (%) of system using features extracted by applying (i) all dependency relations (ii) selective dependency relations (SemEval dataset).

Approach	Aspect category	Precision (%)	Recall (%)	*F*1-score (%)
Features extracted considering all dependency relations	Ambience	73.42	53.21	61.70
	Miscellaneous	65.56	84.98	74.02
	Food	85.00	82.73	83.85
	Price	76.60	50.70	61.02
	Service	74.03	72.15	73.08
Features extracted considering selective dependency relations	Ambience	63.81	61.47	62.62
	Miscellaneous	70.39	91.85	79.70
	Food	87.85	75.67	81.31
	Price	78.43	56.34	65.57
	Service	74.40	79.11	76.69

**Figure 6:**
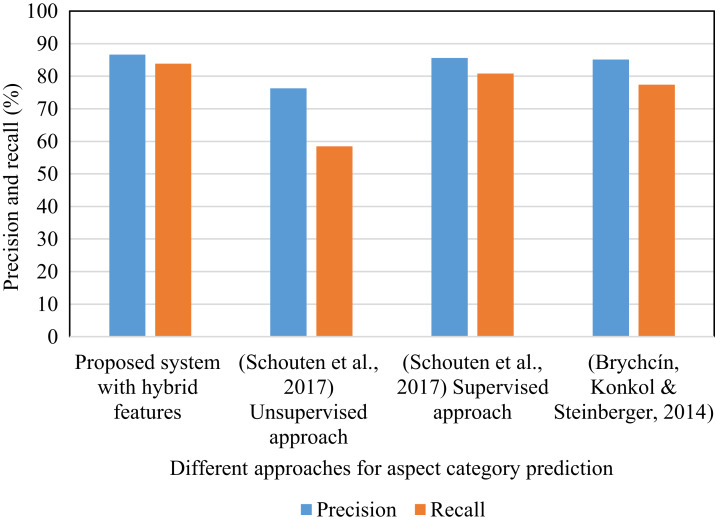
Precision and recall of different methods (SemEval restaurant review dataset).

Table 3 shows the outcomes acquired by utilizing the proposed technique. In it, the hybrid features include lemma based features that are selected using a multivariate filter feature selection method and rule-based features that are extracted by applying selective dependency relations. Table 4 displays that the proposed hybrid system has gained 85.24% *F*1-score which is more than the *F*1-score attained in Schouten et al. (2017) using the supervised and unsupervised approach. The proposed system attained improved results for the food and ambience aspect categories in comparison to the supervised approach in (Schouten et al., 2017). The unsupervised approach in (Schouten et al., 2017) defines the aspect category taking into account the terms in the sentence. This is the unsupervised solution as it prohibits the use of labels and generates a collection of seed terms for each aspect category. Using a semantic lexicon like WordNet, the collection of seed terms for each aspect category is created. Fig. 6 shows the precision and recall values obtained using different methods. These approaches are implemented using the SemEval restaurant review dataset. The precision and recall percentage in the proposed system is improved relative to the other methods. In the proposed system, the feature selection method is applied for the lemma features and the selective dependency relations are considered to select dependency rule based features. This encouraged the relevant features to be picked. In addition to this, the use of correlation helped to prevent feature redundancy. The use of relevant features and the elimination of redundancy resulted in increased percentage of precision and recall.

**Table 3:** *F*1-score (%) in proposed approach using hybrid features (SemEval restaurant review dataset).

Approach	Aspect category	Precision (%)	Recall (%)	*F*1-score (%)
Proposed system with Hybrid approach	Ambience	86.46	76.15	80.98
	Miscellaneous	82.57	77.25	79.82
	Food	88.86	95.13	91.89
	Price	87.69	80.28	83.82
	Service	87.73	90.51	89.10

**Table 4:** *F*1 score (%) obtained using different methods.

Approach	Precision (%)	Recall (%)	*F*1-score (%)
SemEval restaurant review dataset			
Proposed system with hybrid features	86.66	83.86	85.24
(Schouten et al., 2017) Unsupervised approach	76.26	58.48	66.20
(Schouten et al., 2017) Supervised approach	85.58	80.82	83.13
(Brychcín, Konkol & Steinberger, 2014)	85.1	77.4	81.06
Yelp restaurant review dataset			
Proposed system with hybrid features	83.84	81.93	82.88
(Kiritchenko et al., 2014) (Constrained)	86.53	78.34	82.23
(Panchendrarajan et al., 2017)	70.87	95.83	81.48
Patient reviews on drug (Kaggle dataset) (Rachel, 2018)			
Proposed system with hybrid features	76.49	80.47	78.43

**Figure 7:**
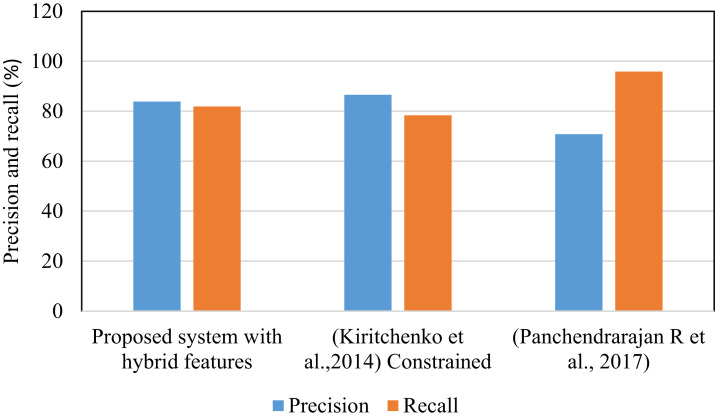
Precision and recall of different methods (Yelp dataset).

Methods cited in Fig. 7 used Yelp restaurant review dataset. Table 4 shows that the proposed system has shown improved results on this dataset compared to other methods. In this work, 2524 reviews are randomly selected from Yelp dataset. From this 1755 are used at the time of training and 769 are used for testing. These results obtained by considering categories like restaurant, ambience, food and service. This algorithm is also tested on Kaggle dataset that includes patient reviews on drugs and the aspect categories are disease name. The system yield *F*1 score 78.43% on Kaggle dataset. For this experimentation, 2100 reviews are randomly selected from Kaggle.

## Conclusions

In this work, two approaches for the aspect category prediction task are proposed. In the first, a multivariate filter approach of feature selection is offered. This shows that the relevant features increase the performance of classification if redundancy among selected features is reduced. In the second approach, dependency relation based features are selected for aspect category prediction. This approach shows that features extracted using selective grammatical rules improve the performance of classification compared to features extracted using all rules. The hybrid feature set is a combination of multivariate filter based lemma features and selective grammatical rule-based features. The use of hybrid feature set increases the *F*1 score of aspect detection task to 85.24%. This work can be further extended by adding semantic features and using a combination of supervised and unsupervised approaches. Also, the feature set can be enhanced by adding bi-tagged features.

## Supplemental Information

10.7717/peerj-cs.347/supp-1 Supplemental Information 1 mysqlDB. Includes aspectmining, aspectmining-yelp, aspectmining-drug for SemEval, yelp and drug dataset, respectively, and the HeidiSQL5.0 tool should be used to view these files.

10.7717/peerj-cs.347/supp-2 Supplemental Information 2 Final dataset. Pre-processed training and testing files of the datasets used in this work.

10.7717/peerj-cs.347/supp-3 Supplemental Information 3 Aspect Based Sentiment Analysis. Includes all Java files related to this work, and NetBeans 8.0.2 IDE should be used to view these files.

10.7717/peerj-cs.347/supp-4 Supplemental Information 4 Raw Dataset—Phase A. SemEval restaurant review dataset.

10.7717/peerj-cs.347/supp-5 Supplemental Information 5 Raw Dataset—Train. SemEval restaurant review dataset.

## Additional Information and Declarations

### Data Availability

The following information was supplied regarding data availability: Data and code are available at GitHub: https://github.com/bhavanaacc/ABSA. SemEval data is available at MetaShare: http://metashare.ilsp.gr:8080/repository/browse/semeval-2014-absa-train-data-v20-annotation-guidelines/683b709298b811e3a0e2842b2b6a04d7c7a19307f18a4940beef6a6143f937f0/. This dataset can be accessed after completing the registration process. After registration using Username and Password, one can access the dataset. Under the distribution licence of the dataset provider, the restriction is that the dataset can be used for “Academic Non-Commercial Use, No Redistribution”. Yelp data is available at Kaggle: https://www.kaggle.com/omkarsabnis/yelp-reviews-dataset. Drug data is also available at Kaggle: https://www.kaggle.com/jessicali9530/kuc-hackathon-winter-2018?select=drugsComTest_raw.csv.
